# A Rare Case of Orthograde Retrieval of Extruded Instrument following Periapical Tissue Healing

**DOI:** 10.1155/2022/2589021

**Published:** 2022-01-25

**Authors:** Ahmed Elhakim, Tarek Mohamed Abd El-Wahab

**Affiliations:** ^1^Department of Endodontics, Faculty of Dentistry, Mansoura University, Mansoura, Egypt; ^2^Department of Conservative Dentistry, Yonsei University College of Dentistry, Seoul, Republic of Korea

## Abstract

The ideal retrieval protocol of separated instruments reverts the case to the initial situation prior to the fracture incidence while preserving the tooth hard tissue and the integrity of the supporting structures. When a patient presented for emergency treatment of tooth #37 diagnosed with acute suppurative apical periodontitis, radiographic examination revealed a fractured instrument extruded into the periapex. The treatment options for retrieval were limited to replantation. The initial emergency treatment which consisted of orthograde pus drain, radicular disinfection, and intracanal calcium hydroxide dressing completely resolved patient's symptoms. The follow-up radiographs revealed an interesting finding: gradual shift in the separated fragment position into the radicular space allowing a successful nonsurgical removal of the broken instrument. In conclusion, the reaction of periodontal tissues to an extruded instrument fragment remaining in situ may be favourable; thus, a risk and benefit analysis approach is essential to fractured instrument retrieval.

## 1. Introduction

The reported rates of endodontic instrument separation during treatment range from 0.4% to 7.4% [1]. This considerable variation in fracture prevalence reflects its complex and multifarious nature. Canal geometry, instrument properties, mode of action, and operator skill are identified as the main determining factors of separation risk [2]. Nevertheless, it is well-acknowledged among practicing specialist that endodontic files frequently separate even with meticulous adherence to the proper parameters of use.

Depending on its location and its relation to the curvature if present and the ability to visualize it by straight line access, management of separated endodontic instruments includes nonsurgical retrieval, bypassing, follow-up, and surgical treatment [3, 4]. Higher rates of treatment failure are associated with unretrieved instruments impeded in canals with preexisting preapical lesions, especially when separations occur earlier in the treatment [5–7]. This is attributed to the residual bacterial biofilms harbored in the artificial isthmus created by the instrument and beyond the point of impedance [3]. When the separated fragment extends beyond the apex due to an improper working length control at the moment of fracture or being pushed during a failed attempt of retrieval, additional complications may arise including injury to vital structures, persistent infection, and foreign body reaction [8].

Fractured instruments extending beyond the apical foramen have a significantly reduced probability of retrieval which explains the noticeable paucity of reports on these cases [3, 4]. The available literature is comprised of single case reports discussing management of partially extruded instrument fragments only, mainly by apical surgery or replantation with few reports describing successful orthograde retrieval [9–13] To the best of our knowledge, this is the first report describing the unusual periodontal tissue reaction to and the successful nonsurgical retrieval of a fractured endodontic instrument completely extruded outside the root canal.

## 2. Case Report

A 30-year-old healthy female patient presented with pain and swelling in the lower left side of the mandible. The pain was severe, disturbed her sleep, and was not relived by medication. She reported a recurrent mild swelling (2 or 3 times annually) in the same area for the past three years.

**Figure 1:**
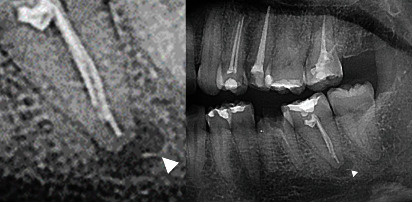
Emergency visit. The patient had severe pain and cellulitis with a fractured instrument beyond the apex (arrowhead). The patient was treated with systemic antibiotic with orthograde pus discharge followed by radicular disinfection and intracanal calcium hydroxide dressing.

The patient had facial asymmetry caused by cellulitis. Intraoral examination revealed a fluctuant swelling on the buccal mucosa adjacent to grossly decayed tooth #36 and filled tooth #37 which was exceptionally sensitive to palpation. The gingival probing depths were normal. Radiographic examination revealed that tooth 37 had a single, poorly obturated canal and a periapical radiolucency with a separated instrument located 3 mm away from the apex (Figure 1).

Tooth 37 was diagnosed as the causative tooth with acute suppurative apical periodontists. After anesthesia and endodontic access, loosely packed root filling was removed the by H-Files (Mani Inc, Tochigi, Japan). Yellow purulent discharge drained through the access cavity and was encouraged by a sterile saline flushed using side vent needle (EndoTop, Cerkamed, Stalowa Wola, Poland). Following working length determination, apical diameter was gauged to reveal a #80 sized wide apical foramen. The canal walls were planned without enlargement using H-files while being irrigated with 2% sodium hypochlorite solution (Chloraxid, Cerkamed, Stalowa Wola, Poland) that was activated ultrasonically using irrisafe-20 (Acteon Satelec, Merignac, France). The canal was then dried and medicated with calcium hydroxide (Metapaste, Meta Biomed, Chungbuk, Korea), and the access was sealed by Cavit (3 M, MN, USA). The unrestorable tooth 36 was extracted, and amoxicillin/clavulanic acid 1 gm tab every 12 hours and Ibuprofen 600 mg tab every 8 hours were prescribed.

After three days, the patient experienced sudden pain exacerbation. The canal was re accessed and purulent discharges exuded once again from the canal. After copious irrigation with sodium hypochlorite and ultrasonic activation, the canal was dried, remedicated with Metapaste, and sealed.

**Figure 2:**
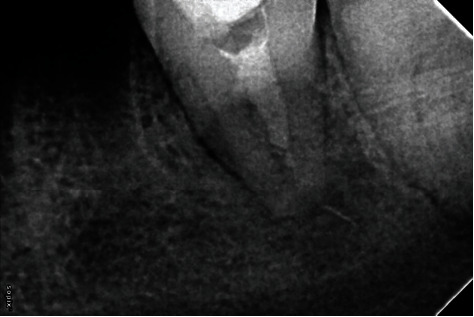
First recall after 8 months. Due to the change in orientation of the broken instrument and the wide apical foramen, a decision was taken to remedicate the canal and follow-up the case as the tooth was functional, and the patient was asymptomatic.

The patient missed the obturation appointment and returned eight months later for an unrelated complaint. She reported complete absence of pain and normal functionality of the treated tooth since the last treatment. Clinical examination revealed an intact temporary restoration, healthy periapical mucosa with normal responses to percussion, and periapical palpation tests. Radiographic examination demonstrated partial bone healing and a shift in the separated fragment position towards the tooth apex (Figure 2).

**Figure 3:**
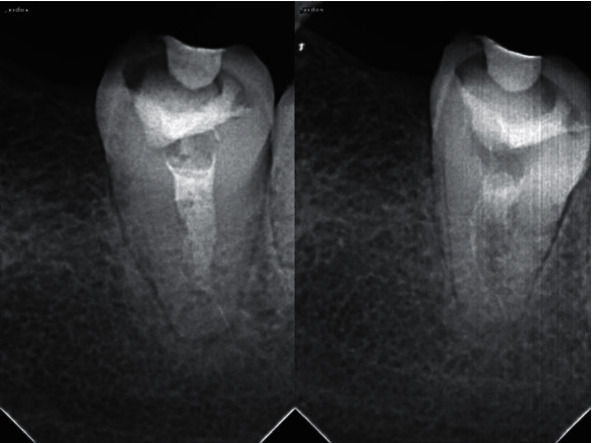
Annual follow-up radiographs demonstrating movement of the fractured segment into the root canal.

**Figure 4:**
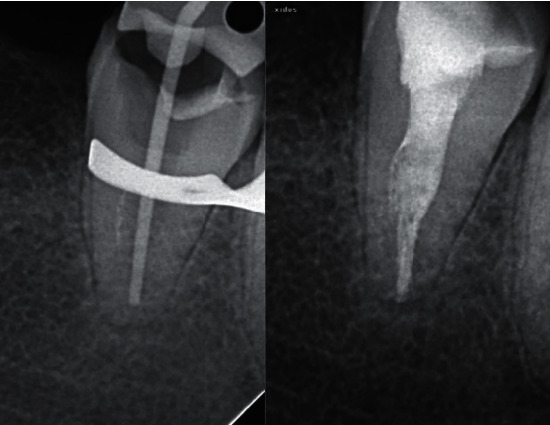
Retrieval of the fractured segment and obturation 32 months after the initial treatment. Patient remains asymptomatic.

The treatment plan changed to a follow-up approach with long-term calcium hydroxide dressing before obturation, to observe the possibility of further movement of the separated fragment. Annual follow-up radiographs demonstrated gradual movement of the broken fragment towards and into the radicular space (Figure 3). Thirty-two months after the initial visit, the entire segment settled inside the root canal, and retrieval attempt using ultrasonically activated irrigant was successful. The canal was dried and obturated with guttapercha and resin sealer (Adseal, Meta Biomed, Chungbuk, Korea) by continuous wave of compaction technique, and the coronal structure was restored using resin composite (Filtek Z350, 3 M ESPE, USA) (Figure 4).

## 3. Discussion

The main goal of managing a separated instrument is to eliminate or minimize its impact on long-term prognosis. Proper and complete disinfection of the radicular spaces after the incidence of instrument separation neutralize its deleterious effect regardless of the outcome of management protocol. Thus, analysis of the risk and benefit and decision-making are integral to separated instrument retrieval [14]. In certain cases, bypassing or follow-up may be preferred over instrument retrieval where excessive removal of dentin to gain access to and free the impeded fragment may compromise the biomechanical integrity of the tooth and lead to perforations [15, 16]. This statement is especially true in cases involving strategic teeth where uncertain prognosis may halt the treatment or lead to undesirable consequences.

Similarly in our case, tooth 37 was initially planned for replantation to curette the periapical area and remove the extruded file segment since apical surgery was not feasible due to the thick buccal bone ridge. However, the inherent risk of replantation-associated resorption could lead to undesirable outcomes including tooth loss and necessity of a later complex prosthodontic restoration. When the periapical lesion demonstrated signs of healing after the emergency visits, the treatment changed from an active intervention to a passive follow-up.

Stainless steel files are corrosion resistant and biologically inert, while NiTi metal alloys are well tolerated by human tissues [17, 18]. Although we cannot determine whether the lesion developed prior to or after the incident of fracture, it is clear that the abscess drainage and radicular disinfection were crucial to initiation of healing and that the remaining fragment did not retard the process of healing. Consequently, an endodontic instrument extruded into the periapex alone may not constitute a sufficient reason of treatment failure [19, 20].

Previous studies concluded that either an inflammatory or isolative reaction (granulation or calcification) takes place around foreign bodies extruded beyond the tooth apex [19, 21]. Smith's observations on the sequestration of fractured roots and bone fragments have conclude that an intermediate layer of inflammatory exudates surrounding the fragment is necessary to facilitate sequestration which may be retreaded in its absence [22]. In the current case, the initial active inflammation and suppuration might have prevented encapsulation of the extruded fragment by granulation tissue and tipped the periradicular tissue reaction towards sequestration, while the wide apical foramen mostly caused by over instrumentation and the unfilled radicular space may have facilitated tissue drainage and file repositioning into the root canal.

## 4. Conclusion

We described the spontaneous retrieval of an instrument fragment extruded into the periapex of tooth 37. Within the limitation of this single study, the findings from this case support the available evidence from the literature of a risk and benefit analysis approach when managing a fractured endodontic instrument. If the source of infection within the radicular spaces has been addressed, the periodontal reaction to an extruded endodontic instrument may be favorable even with maintenance of the fragment in situ. As single observations do not permit any more than a speculation as to the nature of this phenomena and because our understanding of the periodontal tissue reaction to foreign bodies is based on limited classical observations, further studies are needed to elude its governing dynamics and the underlying mechanisms.

## Data Availability

All data generated or analysed during this case are included in this published article.
